# Application of piezoelectric materials in the field of bone: a bibliometric analysis

**DOI:** 10.3389/fbioe.2023.1210637

**Published:** 2023-08-04

**Authors:** Yu-Qin Zhang, Qian Geng, Chao Li, Hai-Cheng Wang, Chuan Ren, Yi-Fan Zhang, Jun-Sheng Bai, Hao-Bo Pan, Xu Cui, Meng-Xuan Yao, Wei Chen

**Affiliations:** ^1^ Department of Orthopaedic Surgery, Third Hospital of Hebei Medical University, Shijiazhuang, Hebei, China; ^2^ Key Laboratory of Biomechanics of Hebei Province, Shijiazhuang, Hebei, China; ^3^ NHC Key Laboratory of Intelligent Orthopaedic Equipment, Shijiazhuang, Hebei, China; ^4^ Center for Human Tissues and Organs Degeneration, Shenzhen Institute of Advanced Technology, Chinese Academy of Sciences, Shenzhen, China

**Keywords:** piezoelectric, PVDF, PHBV, BaTiO3, bone, tissue engineering, bibliometric

## Abstract

In the past 4 decades, many articles have reported on the effects of the piezoelectric effect on bone formation and the research progress of piezoelectric biomaterials in orthopedics. The purpose of this study is to comprehensively evaluate all existing research and latest developments in the field of bone piezoelectricity, and to explore potential research directions in this area. To assess the overall trend in this field over the past 40 years, this study comprehensively collected literature reviews in this field using a literature retrieval program, applied bibliometric methods and visual analysis using CiteSpace and R language, and identified and investigated publications based on publication year (1984–2022), type of literature, language, country, institution, author, journal, keywords, and citation counts. The results show that the most productive countries in this field are China, the United States, and Italy. The journal with the most publications in the field of bone piezoelectricity is the International Journal of Oral & Maxillofacial Implants, followed by Implant Dentistry. The most productive authors are Lanceros-Méndez S, followed by Sohn D.S. Further research on the results obtained leads to the conclusion that the research direction of this field mainly includes piezoelectric surgery, piezoelectric bone tissue engineering scaffold, manufacturing artificial cochleae for hearing loss patients, among which the piezoelectric bone tissue engineering scaffold is the main research direction in this field. The piezoelectric materials involved in this direction mainly include polyhydroxybutyrate valerate, PVDF, and BaTiO3.

## 1 Introduction

Bones are structures in the human body that have many important functions, and have the ability to repair and regenerate themselves after injury. However, the repair and regeneration of bone tissue involves a cascade of complex steps, including osteogenesis, angiogenesis and inflammation (Xu et al., 2023). For large bone defects caused by trauma, infection, and other factors, it is difficult to rely on the repair of bone tissue itself. Therefore, we need to find suitable bone substitute materials that can provide structural support for bone defects and stimulate the potential for bone regeneration, promote cell proliferation, migration, and bone regeneration differentiation (Zhang et al., 2022).

Common bone substitute materials include hydroxyapatite (HA), polylactic acid (PLA), poly (lactic-co-glycolic acid) (PLGA), *etc.* However, these materials cannot simulate the bone microenvironment and stimulate bone formation through electrical, chemical, or other signals. Therefore, we hope to find a bone substitute material that can convert external stimuli into electrical or chemical signals to stimulate bone formation.

Piezoelectric materials are materials that convert mechanical stimulation into electrical stimulation. Electrical stimulation can promote Ca2+ influx or Ca2+ release from the endoplasmic reticulum by changing the distribution of surface receptors on osteoblasts and promoting the opening of calcium ion channels. The elevated Ca2+ in the cytoplasm can activate a large amount of calmodulin, thereby promoting osteoblast proliferation (Brighton et al., 2001). At the same time, electrical stimulation can also affect cells migration through electrotaxis (Isaacson and Bloebaum, 2010). Recruited immune cells regulate the catabolism or anabolism of bone during the formation and remodeling phases through immune responses (Chen et al., 2023; Liu et al., 2023). Accelerating the migration and proliferation of osteoblasts is conducive to the formation of new bone. Therefore, piezoelectric materials have received widespread attention in the field of bone-related materials. In order to study the current research status of piezoelectric materials in the field of bone-related materials, we used bibliometric analysis.

Bibliometric analysis is based on big data analysis, and evaluates key contributors in a field (such as authors, countries, and institutions, *etc.*) quantitatively and visually, and determines their collaborative networks. These results help to analyze the current hottest research directions in a certain field. According to our search in databases such as Web of Science and PubMed, there are currently no bibliometric articles in this field. Our study fills the gap in bibliometric analysis in this field.

We searched for all literature in this field as of 4 December 2022 in the Web of Science database, and downloaded all information of the retrieved literature. We used two software programs, CiteSpace and R language, which are widely used in bibliometric analysis, to explore the collaboration network among published literature, determine their potential relationships, and evaluate the research progress and current main research directions in this field.

## 2 Materials and methods

### 2.1 Literature search

We used the Web of Science (WOS) core collection database (SCI-EXPANDED, SSCI, A&HCI, CPCI-S, CPCI-SSH, ESCI, CCR-EXPANDED and IC) by Clarivate Analytics in Philadelphia, PA, United States to identify relevant articles on piezoelectric materials in bone. Compared to common official websites such as Scopus, the official websites of WoS had less incidence of mislabelled review (Rajabi et al., 2015). WOS data come from journals, books, patents, conference records, web resources (including free and open resources), *etc.* WOS allows extraction of many articles with complete information, including titles, author names, cited references, *etc.* WOS covers citations in scientific publications since 1,900 and includes all high-impact scientific journals. Additionally, this database has two major strengths which are reference tracing and citation reporting, as it enables search within leading academic journals and citation networks and can powerfully trace references and citation activities to explore research outputs in a specific area.

The search formula was [(((((TS=(“bone*”)) OR TI=(“bone*”)) OR AB=(“bone*”)) OR TS=(“condyle*”)) OR TI=(“condyle*”)) OR AB=(“condyle*”)] AND [ALL=(“material*”)] AND [(((TS=(“Piezoelectric*”)) OR TI=(“Piezoelectric*”)) OR AB=(“Piezoelectric*”)) OR AK=(“Piezoelectric*”)].

**FIGURE 1:**
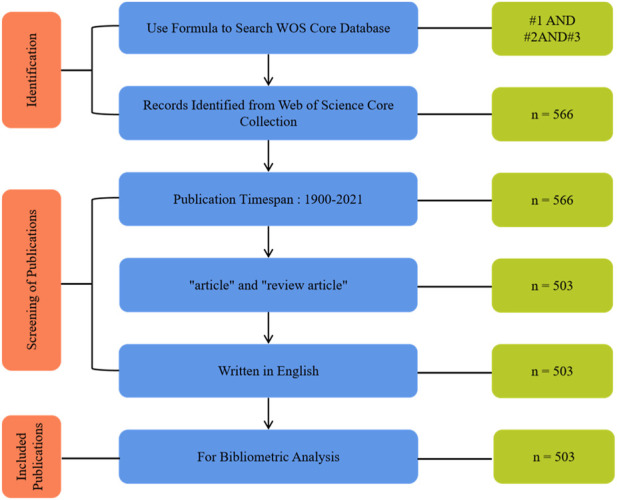
Criteria and flowchart for inclusion and exclusion of studies.

Using the above search formula, a cross-sectional search was conducted on 4 December 2022, and a total of 566 publications were retrieved from WOS. Finally, all available published data were reviewed and evaluated to identify publications that focus on piezoelectric materials in bone. Figure 1 shows the search and inclusion/exclusion process used in this study to identify appropriate publications from the WOS database. Publication types were set to “articles” and “review articles”, and language was set to “English”. In this study, only articles with scientific information conveyors including research articles and reviews are included in our database. The final results of the screening were output to the dataset, including citation information (authors, article title, publication year, source title, volume, issue, page, citation count, source and document type) and reference bibliography information (affiliation, editor, keywords, and funding details).

Since the data used in this study were obtained from public databases and involved no direct interaction with human or animal subjects, ethical approval was not necessary.

### 2.2 Analysis tools

To describe all the literature features related to piezoelectric materials and bones, we further analyzed all qualified data using Bibliometrix (RStudio, V1.4) and CiteSpace V5.8 R3 (Drexel University, Philadelphia, PA, United States).

### 2.3 Bibliometric analysis

The data set was imported into the R package bibliometrix and analyzed for publication trends by year, which were then presented in a line graph. Additionally, R package bibliometrix was used to analyze the annual publication trends of journals, different countries and regions, collaboration between countries/regions, and citation counts. The top 100 high-frequency keywords were outputted as word clouds and ThematicMaps using bibliometrix. ThematicMaps start with a co-occurrence network of keywords and plot the typological themes of a field on a two-dimensional map. The methodology is based on the proposal by Cobo et al. and allows for easier interpretation of the research topics formulated within the framework. The analysis is based on KeyWords Plus (KWP), which are words or phrases that frequently appear in reference titles of papers but not in the paper titles themselves. The process of generating KWP is unique to the Clarivate Analytics database.

CiteSpace, the most popular and widely recognized bibliometric visualization tool, is used to visualize collaboration maps between countries/regions, research institutions, co-authors, and co-cited references, and to calculate keyword bursts. The time frame used is from January 1984 to December 2022, with 1 year per time slice. VOSviewer is used to visualize collaboration and co-authorship among institutions.

## 3 Results

### 3.1 Overview of the data

A total of 566 articles were obtained according to the search strategy, and after further screening, 503 articles met the inclusion criteria. The general characteristics of all included articles are shown in Table 1. The total number of citations for all articles was 11,961, with an average of 23.78 citations per article. Among them, there were 457 research papers (90.9%) and 46 reviews (9.1%). Overall, 63 countries/regions, 700 institutions, 2,133 authors, and 231 journals contributed to the field of piezoelectric materials in bone.

**TABLE 1:** General data information.

Description	Results
MAIN INFORMATION ABOUT DATA	
Timespan	1984:2022
Sources (Journals, Books, etc.)	231
Documents	503
Annual Growth Rate %	10.54
Document Average Age	6.49
Average citations per doc	23.78
References	17,544
DOCUMENT CONTENTS	
Keywords Plus (ID)	1,437
Author’s Keywords (DE)	1,281
AUTHORS	
Authors	2,133
Authors of single-authored docs	12
AUTHORS COLLABORATION	
Single-authored docs	12
Co-Authors per Doc	5.58
International co-authorships %	30.82
DOCUMENT TYPES	
article	436
article; early access	5
article; proceedings paper	16
review	45
review; early access	1

**FIGURE 2:**
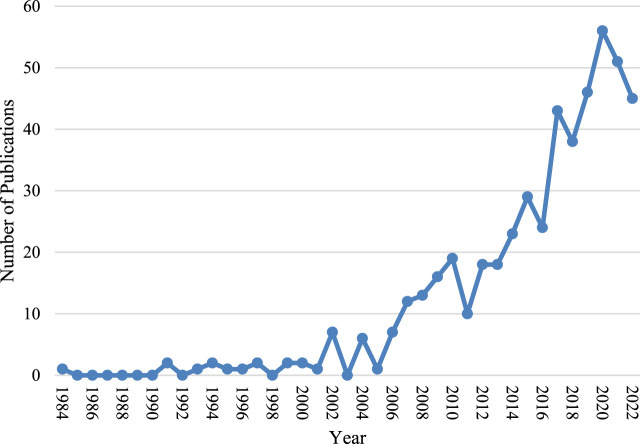
Publication trend from 1984 to 2022.

The number of new articles added to the field of bone piezoelectric materials each year is shown in Figure 2, which shows an overall upward trend with an average annual growth rate of 10.54%. It can be roughly divided into two stages: the first stage was from 1984 to 2006, during which the number of new articles in this field did not exceed 10 per year and the overall trend was stable; the second stage was from 2007 to 2022, during which the number of new articles added each year exceeded 10 and the overall trend was upward.

**FIGURE 3:**
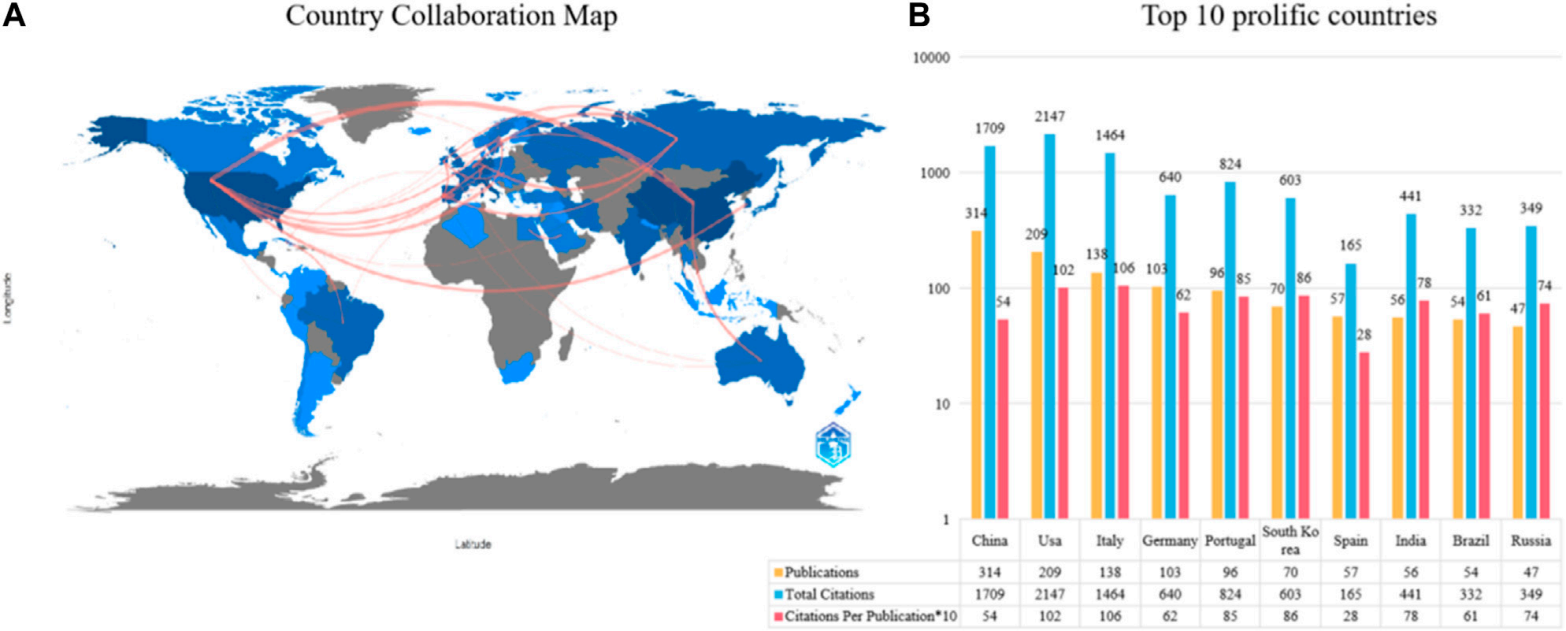
The top 10 most productive countries and international co-operative network. **(A)** Collaboration among prolific countries which published more than 10 papers. Node size represents the number of publication, the width of the link is positively correlated with the strength of cooperation. **(B)** The number of publications, total citations (TC), and citations per publication (CPP) of the top 10 most productive countries/regions.

Figure 3A shows the international cooperation network composed of countries/regions that have published at least ten articles in this field. China and the United States are not only the countries with the highest number of total published articles but also actively participate in cooperation with other countries/regions. Figure 3B lists the top ten countries that have made the greatest contributions in this field. China ranks first with 314 articles, followed by the United States with 209 articles, and Italy with 138 articles. In terms of citation, the United States has the highest TC (TC = 2,147), but CPP (CPP = 102) ranks second; China ranks second in TC (TC = 1709), and ninth in CPP (CPP = 54); Italy ranks third in TC (TC = 1,464) and first in CPP (CPP = 106).

### 3.2 Authors and institutions

**FIGURE 4:**
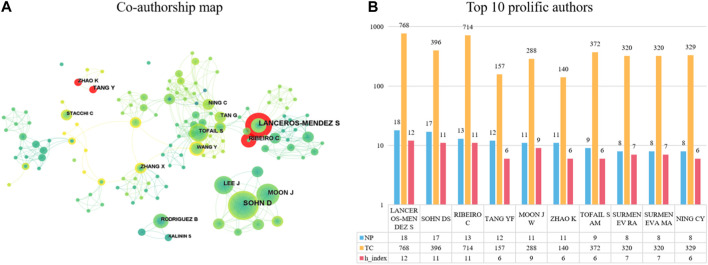
The top 10 most productive authors and collaboration network centered around these authors. **(A)** Collaboration among prolific authors. Node size reflects the number of publications. The width of links represents the cooperative strength. **(B)** The number of publications, total citations (TC) and h-index in the top 10 prolific authors.

Figure 4B shows the top ten authors ranked by the number of publications. The author with the highest number of published articles is Lanceros-Méndez S with 18 publications, followed by Sohn D. S (17 publications) and Ribeiro C (13 publications). In addition, Lanceros-Méndez S is also the most cited author, with 768 citations to their articles. Furthermore, the h-index contribution of this author is also the largest, at 12. The authors with the second and third most citations are Ribeiro C (714 citations) and Sohn D. S (396 citations), with an h-index of 11 for both.

It is worth noting that the co-authorship map analysis in Figure 4A shows that there are six clusters of authors who have a more obvious collaboration network among them. The largest group includes authors such as “Tan G, Ning C, Tofail S, Wang Y,” who collaborate frequently with each other. Additionally, the red annual rings in Figure 4A indicate the active authors in recent years, showing that Lanceros-Méndez S, Ribeiro C, Zhao K, and Tang Y have been very active in recent years.

**FIGURE 5:**
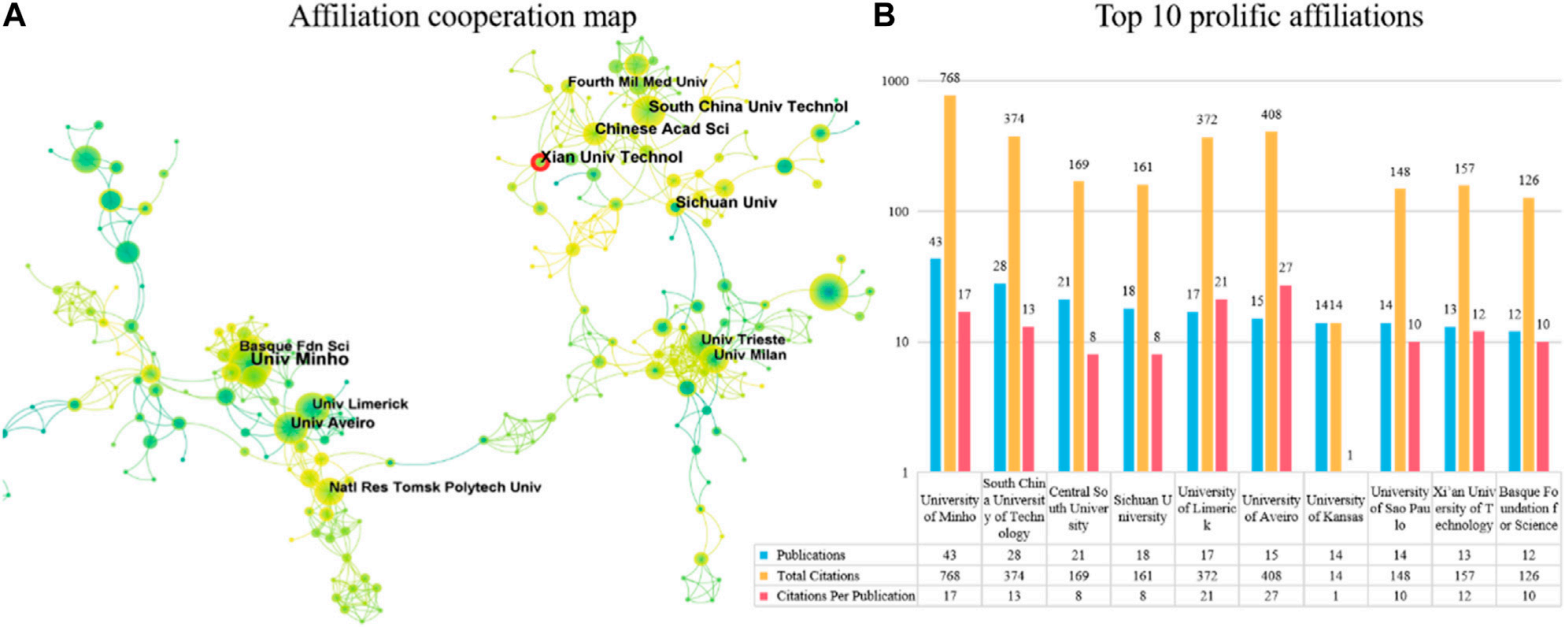
The top 10 most productive affiliations and collaboration network centered around these affiliations. **(A)** Collaboration among prolific affiliations. Node size reflects the number of publications. The width of links represents the cooperative strength. **(B)** The number of publications, total citations (TC), and citations per publication (CPP) in the top 10 prolific affiliations.

In terms of publication ranking, Figure 5B lists the top 10 productive institutions. The institution with the highest number of publications is the University of Minho (n = 43), followed by South China University of Technology (n = 28) and Central South University (n = 21). The institution with the most cited articles is the University of Minho (n = 768), followed by the University of Aveiro (n = 408) and South China University of Technology (n = 374). The institution with the highest average number of citations per article is the University of Aveiro (n = 27), followed by the University of Limerick (n = 21) and the University of Minho (n = 17). Four of the top 10 most productive institutions are located in China.

The co-authorship map analysis of institutions in Figure 5A shows a network of cooperative relationships formed by related institutions in the published articles, with three clusters being more obvious. This indicates that they have close cooperative relationships. Institutions such as South China University of Technology, Chinese Academic Science, and Xian University Technology in China have close cooperation. The red annual rings in Figure 5A indicate the active institutions in recent years, showing that Xian University Technology has been more active in recent years. Additionally, institutions such as the University of Trieste and the University of Milan in Italy cooperate closely, while the remaining group includes the University of Limerick in Ireland, the University of Aveiro in Portugal, and the Basque Foundation for Science in Spain.

### 3.3 Journals

Table 2 lists the top 10 productive and co-cited journals. International Journal of Oral & Maxillofacial Implants (IF (2021) = 2.912, Q2) is the most productive journal, with 20 publications (17.7% of the total), followed by Implant Dentistry (IF (2021) = 3, Q2) with 17 publications, and Journal of Oral and Maxillofacial Surgery (IF (2021) = 2.136, Q4) with 16 publications. In terms of citation and impact, International Journal of Oral & Maxillofacial Implants ranks first (381 TC, 11 h-index), followed by Implant Dentistry (TC = 340, h-index = 11) and Journal of Oral and Maxillofacial Surgery (TC = 308, h-index = 10). However, the number of publications may not necessarily represent their impact on a given field. Therefore, we used VOSviewer to identify the co-cited journals frequently cited in the field of bone piezoelectric materials. The top 3 co-cited journals are Biomaterials (cited 224 times), Acta Biomaterialia (cited 152 times), and Nature (cited 139 times).

**TABLE 2:** Top 10 prolific journals and co-cited journals.

Rank	Journal	Publications	Total citations	h index	If (2021)	Co-cited journal	Co-citations	If (2021)
1	International Journal Of Oral & Maxillofacial Implants	20	381	11	2.912	Acta Biomaterialia	152	10.633
2	Implant Dentistry	17	340	11	3.000	Biomaterials	224	15.304
3	Journal Of Oral And Maxillofacial Surgery	16	308	10	2.136	Nature	139	69.504
4	Acs Applied Materials & Interfaces	13	321	9	10.383	Journal Of Biomedical Materials Research Part A	129	4.854
5	Clinical Implant Dentistry And Related Research	11	297	9	4.259	Journal Of The Physical Society Of Japan	118	1.933
6	Clinical Oral Implants Research	8	270	7	5.021	J Biomed Mater Res	117	-
7	Acta Biomaterialia	6	622	6	10.633	Journal Of Biomechanics	115	2.789
8	Advanced Materials	6	486	6	32.086	Materials Science & Engineering C-materials For Biological Applications	114	-
9	Colloids And Surfaces B-Biointerfaces	7	432	6	5.999	Journal Of Applied Physiology	113	3.88
10	Journal Of Biomedical Materials Research Part B-Applied Biomaterials	9	152	6	3.405	Science	111	63.714

### 3.4 Most cited and co-cited papers

**FIGURE 6:**
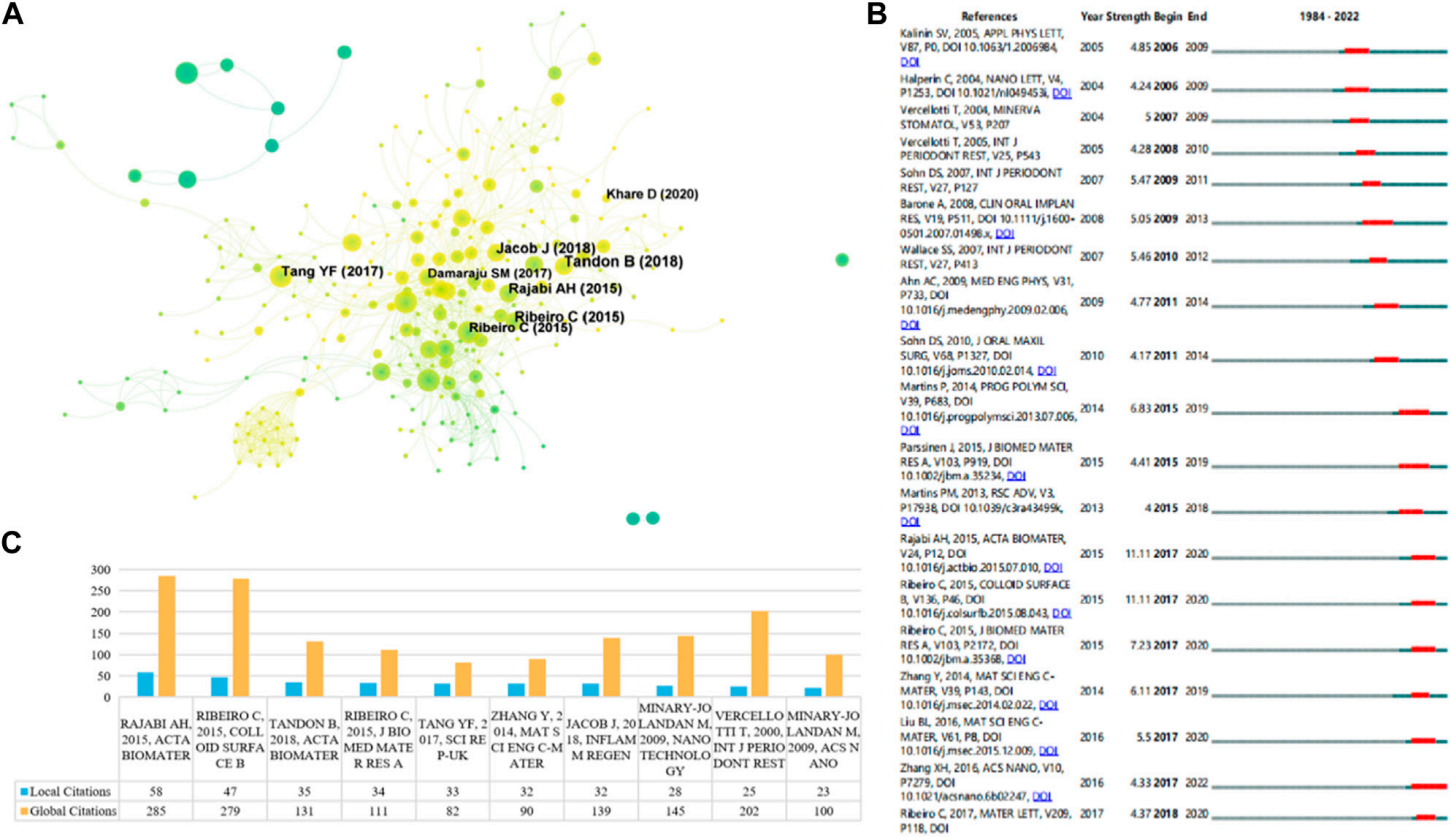
**(A)** Reference co-citation network. The nodes and links are distinguished by colors, in which blue reflects an earlier co-citation relationship, yellow represents a recent co-citation relationship. The size of nodes is positively associated with the citation number. **(B)** The top 19 co-cited references with the strongest citation bursts represent important publications in different periods. The red bar indicates the burst duration. The burst strength suggests the importance to the research field. **(C)** The top 10 publications with the most local citations (LC) and most global citations (GC).

In order to identify the most influential research in this field, we used R language and CiteSpace to extract the top 10 most cited and co-cited papers. Table 3 and Figure 6C list the top 10 most cited papers in this field, including 6 original articles and 4 reviews. The most cited paper (LC = 58, GC = 285) was published by Amir Hossein Rajabi in Acta Biomaterial in 2015, titled “Piezoelectric materials for tissue regeneration: A review.” In this review, the author summarized the applications of piezoelectric materials in different biological tissues, the mechanism of electrical stimulation affecting cellular responses, and the latest developments in the preparation and application of piezoelectric scaffolds (Yeung, 2019). In addition, three other reviews reported on similar topics (Ribeiro et al., 2015a; Jacob et al., 2018; Tandon et al., 2018). One research article reported a case of piezoelectric surgical knife used in dental surgery in 2000 (Vercellotti, 2000). Two research articles measured the piezoelectric coefficient of type I collagen fibers in bovine Achilles tendon in 2009 (Minary-Jolandan and Yu, 2009a; Minary-Jolandan and Yu, 2009b). One research article reported the promotion effect of piezoelectric material polyvinylidene fluoride (PVDF) on osteogenic differentiation of human adipose-derived stem cells (hASCs) in 2015 (Ribeiro et al., 2015b). Two other research articles reported on the mechanical properties, porosity, pore size, piezoelectric properties, biocompatibility, and promotion effect on osteogenic differentiation of hydroxyapatite and BaTiO3 mixed piezoelectric bone substitute materials in 2014 and 2017, respectively (Zhang et al., 2014; Tang et al., 2017). Figure 6A reflects the evolution of the knowledge network in this field. Papers that received more attention in the early years are marked in deep blue, while those that have been mainly focused on in recent years are marked in yellow. Figure 6B shows the top 19 papers with the most citation bursts, where two papers have the highest citation bursts (n = 11.11), one is the review paper by Amir Hossein Rajabi published in Acta Biomaterial in 2015 (Yeung, 2019), and the other is the paper titled “Piezoelectric polymers as biomaterials for tissue engineering applications” published in Colloid and Surface B-Biointerfaces in 2015 (Ribeiro et al., 2015a). The paper that had the highest citation burst and ended in 2022 was the one published in ACS Nano in 2016 titled “Nanocomposite Membranes Enhance Bone Regeneration Through Restoring Physiological Electric Microenvironment” (Minary-Jolandan and Yu, 2009b).

**TABLE 3:** General data information.

Publications	Year	Journal	References
Piezoelectric materials for tissue regeneration: A review	2015	Acta Biomaterial	Yeung (2019)
Piezoelectric polymers as biomaterials for tissue engineering applications	2015	Colloid and Surface B-Biointerface	Ribeiro et al. (2015a)
Piezoelectric materials as stimulatory biomedical materials and scaffolds for bone repair	2018	Acta Biomaterial	Tandon et al. (2018)
Dynamic piezoelectric stimulation enhances osteogenic differentiation of human adipose stem cells	2015	Journal of Biomedical Materials Research Part A	Ribeiro et al. (2015b)
Fabrication and *in vitro* biological properties of piezoelectric bioceramics for bone regeneration	2017	Science Report	Tang et al. (2017)
Aligned porous barium titanate/hydroxyapatite composites with high piezoelectric coefficients for bone tissue engineering	2014	Materials Science & Endineering C-Materials for Biological Applications	Zhang et al. (2014)
Piezoelectric smart biomaterials for bone and cartilage tissue engineering	2018	Inflammation and Regeneration	Jacob et al. (2018)
Nanoscale characterization of isolated individual type I collagen fibrils: polarization and piezoelectricity	2009	NanoTechnology	Minary-Jolandan and Yu (2009a)
Piezoelectric surgery in implantology: A case report - A new piezoelectric ridge expansion technique	2000	International Journal of Periodontics & Restorative Dentistry	Vercellotti (2000)
Uncovering Nanoscale Electromechanical Heterogeneity in the Subfibrillar Structure of Collagen Fibrils Responsible for the Piezoelectricity of Bone	2009	ACS NANO	Minary-Jolandan and Yu (2009b)

### 3.5 Keyword analysis

**FIGURE 7:**
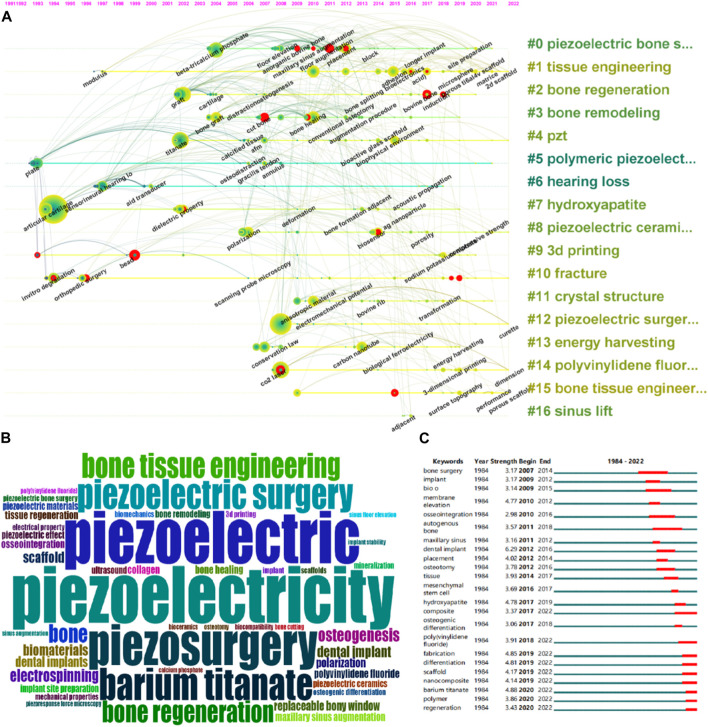
Co-occurrence analysis of keywords. **(A)** The co-occurrence networks of author keywords were visualized by VOSviewer. Large nodes represent keywords with relative higher occurrence; Same color indicates relatively closer relationship; **(B)** Wordcloud of the collection of literature keywords included in the study. **(C)** The top 23 keywords with the strongest citation bursts represent hot topics in different periods. The red bar indicates the burst duration. The burst strength refers to the importance to the research field.

Keyword co-occurrence clustering analysis and keyword burst detection (CiteSpace) were used to identify research hotspots and emerging topics. Figure 7A shows the keyword clustering results in CiteSpace, with a total of 17 categories. Figure 7B shows the keyword cloud. Figure 7C lists the top 23 keywords in terms of burst strength, sorted by their active time, where the red segment indicates the active time of the keyword. The keyword with the highest burst strength is “barium titanate” (n = 4.88), which was active from 2020 to 2022. Keywords that were active until 2022 include “composite” (n = 3.27), “fabrication” (n = 4.85), “poly (vinylidene fluoride) (PVDF)” (n = 3.91), “scaffold” (n = 4.17), “differentiation” (n = 4.81), “nanocomposite” (n = 4.14), “polymer” (n = 3.86), and “regeneration” (n = 3.43).

## 4 Discussion

### 4.1 Overall situation

Based on the results of this study, it can be seen from the trend of annual publication volume that this field has received extensive attention from researchers since 2007 (Figure 2). The global trend and research hotspots of application of piezoelectric materials in the field of bone are mainly concentrated in North America, Europe, and Asia (Figure 3A). Institutions in these regions also have a strong influence in terms of research collaboration and number of published papers.

### 4.2 Author and institutions

Regarding the authors, the top 10 most prolific authors are listed, and their information including the number of published papers, total citation, and H-index. Among them, LANCEROS-MENDEZ S and RIBEIRO C are in the same cluster, it means they had closely cooperation. They all came from Minho University in Portugal, with 18 and 13 published papers respectively, and have received high total citation counts and H-index (Figure 4B). Besides, they have been active in research in this field (Figure 4A). We believe that the articles they have co-authored in recent years have a certain guiding effect on the latest and hottest research directions in this research field. In Korea, SOHN DS is from Catholic University of Daegu, published 15 papers, with a total citation count of 396 times and an H-index of 11. In China, Zhao K and Tang YF are from Xi’an University of Technology in China. They have been very active in this field in recent years (Figure 4A), and have made outstanding contributions in the field of application of piezoelectric materials in the field of bone. Xi’an University of Technology in China also have been very active in this field in recent years (Figure 5A). Thus, we believe that the articles they have co-authored in recent years have a certain guiding effect on the latest and hottest research directions in this research field. The research achievements of these authors have important significance for the development of application of piezoelectric materials in the field of bone.

As shown in Table 4, We selected articles co-authored by LANCEROS-MENDEZ S and RIBEIRO C in the past 5 years, and summarized the research content of the papers. These literature focus on piezoelectric materials for biomedical and tissue engineering applications, including polymer piezoelectric materials (PVDF, PHBV) and magnetically active piezoelectric materials (by incorporating magnetostrictive Terfenol-D into piezoelectric materials). The research content involves 1) the design, synthesis and osteogenic performance of piezoelectric bone tissue engineering scaffolds. 2) the effect of electrical stimulation generated by piezoelectric materials on cell growth and differentiation. 3) the piezoelectricity generated by piezoelectric materials The regulation of bacterial growth and differentiation by electrical stimulation. 4) What role does surface charge play in the interaction between cells and piezoelectric material scaffolds. 5) Adjust the overall biological and physical characteristics of tissue engineering scaffolds by incorporating different other substances.

**TABLE 4:** Senentxu Lanceros-Méndez and Ribeiro Clarisse’s collaborative article.

Publications	Piezoelectric material	Other	References
Fluorinated Polymers as Smart Materials for Advanced Biomedical Applications	piezoelectric poly (vinylidene) fluoride (PVDF)		Cardoso et al. (2018)
Electroactive poly (vinylidene fluoride)-based structures for advanced applications	PVDF		Ribeiro et al. (2018)
T ailored Biodegradable and Electroactive Poly (Hydroxybutyrate-Co-Hydroxyvalerate) Based Morphologies for Tissue Engineering Applications	hydroxybutyrate-co-hydroxyvalerate (PHBV)	Hydroxybutyrate-Co-Hydroxyvalerate (CFO)	Amaro et al. (2018)
Tailoring Bacteria Response by Piezoelectric Stimulation	PVDF		Carvalho et al. (2019)
Bioinspired Three-Dimensional Magnetoactive Scaffolds for Bone Tissue Engineering	PVDF	CoFe 2 O 4	Fernandes et al. (2019)
Surface Charge-Mediated Cell−Surface Interaction on Piezoelectric Materials	PVDF		Ribeiro et al. (2020a)
Silica nanoparticles surface charge modulation of the electroactive phase content and physical-chemical properties of poly (vinylidene fluoride) nanocomposites	PVDF		Ribeiro et al. (2020b)
Morphology Dependence Degradation of Electro- and Magnetoactive Poly (3-hydroxybutyrate-co-hydroxyvalerate) for Tissue Engineering Applications	PHBV	Fe3O4	Amaro et al. (2020)
Magnetic Bioreactor for Magneto-, Mechano- and Electroactive Tissue Engineering Strategies	PVDF	magnetostrictive Terfenol-D (TD)	Castro et al. (2020)
Patterned Piezoelectric Scaffolds for Osteogenic Differentiation	PVDF		Marques‐Almeida et al. (2022)
Understanding Myoblast Differentiation Pathways When Cultured on Electroactive Scaffolds through Proteomic Analysis	PVDF		Ribeiro et al. (2022)
Two- and three-dimensional piezoelectric scaffolds for bone tissue engineering	PVDF		Silva et al. (2022)
Development and evaluation of different electroactive poly (vinylidene fluoride) architectures for endothelial cell culture	PVDF		Durán-Rey et al. (2022)
Piezoelectric and Magnetically Responsive Biodegradable Composites with Tailored Porous Morphology for Biotechnological Applications	PHVB	Fe3O4	Marques-Almeida et al. (2022)

As shown in Table 5, We selected articles co-authored by Zhao K and Tang YF in the past 4 years, and summarized the research content of the papers. These literature mainly focus on the preparation, performance and application of bio-piezoelectric ceramics (BaTiO3) and composite materials based on bio-piezoelectric ceramics in biomedical applications. Research covers materials synthesis, characterization, mechanical properties, biocompatibility, and tissue engineering applications. The research contents include: 1) Inducing mineralization and enhancing corrosion resistance by preparing bio-piezoelectric ceramic coatings on titanium alloys. 2) Adjust the physical and biological characteristics of biopiezoelectric ceramic bone tissue engineering scaffolds by incorporating other materials. 3) Enhance the osteogenic performance of bone cement by incorporating bio-piezoelectric ceramic particles into bone cement. 4) Effects of biopiezoelectric ceramic bone tissue engineering scaffolds on cell growth and differentiation.

**TABLE 5:** Zhao, Kang and Tang yufei’s collaborative article.

Publications	Material	Form	References
Fabrication and induced mineralization of bio-piezoelectric ceramic coating on titanium alloys	BaTiO3,TiO2	coating	Tang et al. (2020a)
Graphene/barium titanate/polymethyl methacrylate bio-piezoelectric composites for biomedical application	BaTiO3,PMMA, Graphene	Bone cement	Tang et al. (2020b)
An investigation of the electrical, mechanical and biocompatibility properties of barium titanate/hydroxyapatite bulk ceramics	BaTiO3,HA	composite scaffolds	Jiao et al. (2020a)
Degradation behaviour of non-sintered graphene/barium titanate/magnesium phosphate cement bio-piezoelectric composites	BaTiO3,MPC	Bone cement	Tang et al. (2020c)
Erbium-doped barium titanate/hydroxyapatite composites with enhanced piezoelectric and biological properties	BaTiO3,HA,Er3+	composite scaffolds	Wu et al. (2020a)
Synthesis and properties of porous piezoelectric BT/PHBV composite scaffold	BaTiO3,PHBV	composite scaffolds	Jiao et al. (2020b)
Enhanced corrosion resistance of bio-piezoelectric composite coatings on medical magnesium alloys	BaTiO3,Graphene, MgO,KH2PO4, Na2B4O7·10H2O	Bone cement	Tang et al. (2020d)
*In situ* synthesis of TiO2@ BaTiO3 coaxial nanotubes coating on the titanium surface	BaTiO3,TiO2	coating	Wu et al. (2020b)
Rapid apatite induction of polarized hydrophilic HA/PVDF bio-piezoelectric coating on titanium surface	PVDF,HA	coating	Wu et al. (2021a)
Enhanced compressive strengths and induced cell growth of 1-3-type BaTiO3/PMMA bio-piezoelectric composites	BaTiO3,PMMA	Bone cement	Tang et al. (2021)
Novel dam-like effect based on piezoelectric energy conversion for drug sustained release of drug-loaded TiO2 @ BaTiO3 coaxial nanotube coatingElectroactive Tissue Engineering Strategies	BaTiO3,TiO2	coating	Wu et al. (2021b)
Improved hydrophilicity and durability of polarized PVDF coatings on anodized titanium surfaces to enhance mineralization ability	PVDF	coating	Wu et al. (2021c)

### 4.3 Journals and most cited papers

The journals with the most publications in this field are International Journal of Oral & Maxillofacial Implants, Implant Dentistry, and Journal of Oral and Maxillofacial Surgery, indicating that authors in this field tend to submit to these journals when publishing articles. However, the journals where articles are frequently published in this field do not necessarily have the most comprehensive knowledge in this field. Therefore, we analyzed the commonly cited journals in this field, and the top three journals in terms of citations are Biomaterials, Acta Biomaterialia, and Nature, indicating that these three journals are the most authoritative in the field.

Among the top 10 most cited articles, the content of the reviews focuses on the mechanism and application of piezoelectric materials in bone tissue engineering. This indicates that piezoelectric bone tissue engineering scaffolds are the main research direction in this field. Among the six research articles, only one article is related to piezoelectric surgery, two articles are related to the physical properties of piezoelectric materials, and three articles are related to piezoelectric bone tissue engineering scaffolds. This proves that piezoelectric bone tissue engineering scaffolds are the main research direction in this field.

Among the top ten most cited articles, 4 reviews mainly introduced the application of piezoelectric materials in the field of tissue regeneration and tissue engineering. It covers the role of biological piezoelectric ceramics and polymer piezoelectric materials in bone repair, the effect of piezoelectric stimulation on cell growth and differentiation, the potential of piezoelectric materials in bone and cartilage tissue engineering, *etc.* These reviews provide a theoretical basis for the application of piezoelectric materials in the fields of tissue engineering and regenerative medicine. Six research articles mainly focused on the application and effect of piezoelectric stimulation in bone tissue engineering. In addition, the piezoelectric properties of type I collagen fibers were also deeply explored, which is of great significance for understanding the piezoelectric properties of bone.

### 4.4 Keywords

As shown in Figure 7, the keywords are divided into 17 categories. Cluster #0 includes keywords such as β-tricalcium phosphate, floor elevation, anorganic bovine bone, maxillary sinus augmentation, floor augmentation, placement, block, longer implant, *etc.* It can be found that this cluster of keywords revolves around research on piezoelectric bone surgery in oral and maxillofacial surgery. Cluster #1 includes keywords such as modulus, adhesion bioelectronics acid, microsphere, porous ti6al4v scaffold, matrice, 2 days scaffold, *etc.* It can be found that this cluster of keywords revolves around the preparation of porous piezoelectric tissue engineering scaffold using microspheres. Cluster #2 includes keywords such as graft, cartilage, distraction osteogenesis, bone splitting, bovine bone, induction, *etc.* This cluster also revolves around research on piezoelectric bone surgery in oral and maxillofacial surgery. Cluster #3 includes keywords such as bone graft, cut bone, bone healing, conventional osteotomy, augmentation procedure, *etc.* It can be found that this cluster revolves around the influence of piezoelectric materials on bone remodeling. Cluster #4 includes keywords such as titanate, calcified tissue AFM, bioactive glass scaffold, biophysical environment, *etc.* This cluster mainly revolves around orthopedic implant supports made by mixing piezoelectric materials with bioactive glass or titanate. Cluster #5 includes keywords such as plate, osteodistraction, gracilis tendon, annulus, *etc.*, and this cluster is related to lower limb bone traction surgery implants. Cluster #6 includes keywords such as sensorineural hearing, aid transducer, acoustic propagation, *etc.*, and this cluster mainly revolves around the manufacture of artificial cochlear implants for hearing loss patients. Cluster #7 includes keywords such as articular cartilage, dielectric property, deformation, bone formation adjacent, Ag nanoparticle, *etc.*, and this cluster mainly revolves around adjusting the overall dielectric properties of the support by mixing piezoelectric materials with substances such as Ag nanoparticles. Cluster #8 includes keywords such as polarization, biosensor, porosity, and this cluster mainly revolves around piezoelectric ceramic scaffold. Cluster #9 includes keywords such as bead, sodium potassium, *etc.*, and this cluster mainly revolves around using piezoelectric materials as anti-corrosion coatings on the surface of alloy implants. Cluster #10 includes keywords such as *invitro* degradation, orthopedic surgery, scanning probe microscopy, *etc.*, and this cluster mainly revolves around research on piezoelectric bone surgery in oral and maxillofacial surgery. Cluster #11 includes keywords such as anisotropic material, electromechanical potential, bovine rib, transformation, *etc.* This cluster mainly revolves around the crystal structure of piezoelectric materials. Cluster #12 includes keywords such as curette, *etc.* This cluster mainly revolves around research on piezoelectric bone surgery in oral and maxillofacial surgery. Cluster #13 includes keywords such as conservation law, carbon nanotube, biological ferroelectricity, energy harvesting, *etc.*, and this cluster mainly revolves around the mechanism of energy harvesting. Cluster #14 includes keywords such as CO2 laser, 3-dimensional printing, dimension, *etc.* This cluster mainly revolves around 3D printing of PVDF orthopedic implants. Cluster #15 includes keywords such as surface topography, performance, porous scaffold, *etc.*, and this cluster mainly revolves around the effect of piezoelectric porous scaffolds on bone tissue engineering. Cluster #16 contains adjacent and other related keywords and primarily focuses on research related to sinus lift in Piezoelectric Bone Surgery in oral surgery.

Overall, the 17 clusters are centered around five research directions. The first research direction mainly focuses on Piezoelectric Bone Surgery in oral surgery, with #0, #2, #10, #12, and #16 falling under this category. The second research direction mainly focuses on piezoelectric bone tissue engineering scaffold, with #1, #4, #7, #8, #14, and #15 falling under this category. The third research direction is the manufacturing of artificial cochlear implants for hearing loss patients, with #6 falling under this category. The fourth research direction mainly focuses on the physical properties of piezoelectric materials, with #3, #11, and #13 falling under this category. The fifth research direction mainly focuses on using piezoelectric materials as anti-corrosion coatings on alloy implant surfaces, with #5 and #9 falling under this category.

As shown in Figure 7, the 23 hottest keywords, whose popularity continued into 2022, include composite, fabrication, poly (vinylidene fluoride) (PVDF), scaffold, differentiation, nanocomposite, polymer, and regeneration. These keywords all fall under the second research direction, which demonstrates that piezoelectric bone tissue engineering scaffold is the newest and hottest direction in this field.

### 4.5 Main classification of piezoelectric materials

After the above research, we found that piezoelectric materials are often used in bone-related fields to prepare piezoelectric bone tissue engineering scaffold by 3D printing and non-3D printing, anti-corrosion piezoelectric coatings on alloy implant surface, etc.,. The piezoelectric materials used are mainly divided into two categories, namely, bioceramic piezoelectric materials and polymer piezoelectric materials. In the following, we will summarize the characteristics of these two types of piezoelectric materials respectively.

#### 4.5.1 Bioceramic piezoelectric materials

Piezoelectric ceramics can be used as orthopedic implant materials to mimic the electrical activity of natural bone (Dubey et al., 2019). Multifunctional electroactive perovskites, such as CaTiO3, BaTiO3, *etc.*, are considered as promising bone substitutes (Dubey et al., 2019). Recent advancements in bone tissue engineering have highlighted the potential of piezoelectric materials, particularly Barium Titanate (BaTiO3), in promoting bone formation and osseointegration (). Barium Titanate (BaTiO3) is a commonly used piezoelectric material in biomedical applications due to its high piezoelectric coefficient, biocompatibility, and osteogenic activity.

In several studies, BaTiO3 has been coated on various substrates like ceramics and titanium alloys to create materials that respond to dynamic forces, such as those from Low-Intensity Pulsed Ultrasound (LIPUS) (). These piezoelectric materials have shown to enhance cell viability, adhesion, and osteogenic differentiation, thus promoting osteogenesis ().

Perovskite ceramics, such as (Ba,Ca)TiO3, have been investigated as potential materials for orthopedic applications due to their good biocompatibility and piezoelectric properties (Ahmadi et al., 2019; Dubey et al., 2019; Poon et al., 2020). These ceramics have been shown to promote osteogenic activity and bone formation both *in vitro* and *in vivo* (Ahmadi et al., 2019; Dubey et al., 2019; Poon et al., 2020). In addition, (Ba,Ca) (Zr,Ti)O3 ceramics have been evaluated for their biocompatibility as bone replacement materials (Poon et al., 2020).

Interestingly, studies have shown that these materials not only function at an initial cell culture stage but also show promising results in the long-term by allowing for osseointegration and osteoinduction (Wu et al., 2021c). This has opened up new possibilities in oral implantology and the repair of large bone defects, with the aim to restore the physiological environment of bone tissue ().

The piezoelectric effect has been investigated for its role in enhancing the benefits of 3D hydrogel encapsulation, growth factor delivery, and scaffold modification. These combined effects have shown to improve cell in-growth, greater scaffold pore occupancy by bone tissue, superior vascularization, and an upregulated level of osteogenic proteins (Tang et al., 2020a).

One of the main concerns in the application of piezoelectric materials has been the potential toxicity of particles released from these materials. However, studies have shown that BaTiO3-based piezoelectric materials do not induce any systemic toxicity response or significant inflammatory reaction, thus suggesting their safety in biomedical applications (Yafei et al., 2021).

Despite these promising findings, further research is needed to optimize parameters and evaluate the effects of piezoelectric materials with other types of scaffold materials and cell.

#### 4.5.2 Polymer piezoelectric materials

The application of polymer piezoelectric materials, such as polyvinylidene fluoride (PVDF) and polyhydroxybutyrate valerate (PHBV), in bone tissue engineering has shown promising results in recent studies. These piezoelectric polymers exhibit excellent biocompatibility, making them attractive materials for the construction of functional scaffolds for tissue engineering applications (Li et al., 2019).

The piezoelectric nature of native bone has been a focus of research, with PVDF being a popular piezoelectric polymer due to its ease of processability and good biocompatibility (Gong et al., 2022). The application of a novel piezoelectric actuator for orthopedic application using the converse piezoelectric effect has demonstrated the potential to effectively stimulate bone growth (Reis et al., 2012).

The interaction between piezoelectric PVDF membranes with different surface polarities and the macrophage response has been investigated, showing that surfaces with different charges can modulate the macrophage-immune-osteogenic microenvironment. Negatively charged PVDF promoted the osteogenic differentiation of rat bone marrow mesenchymal stem cells, while positively charged PVDF demonstrated pro-inflammatory properties without compromising subsequent osteogenesis (Zhu et al., 2022).

In addition to pure PVDF, composite materials have also shown promise in the field of bone tissue engineering. The optimal conditions for electrospinning of composite fibers were determined from a combination of poly-capro-lactone (PCL), poly-vinylidene fluoride (PVDF), and hydroxyapatite (HA). The PVDF/PCL/HA scaffold showed an approximate 20% increase in piezoelectric properties compared to a PCL scaffold and revealed significant improvement in cell adhesion and function (Peidavosi et al., 2022).

The use of piezoelectric PVDF foam constructed via solid-state shear milling and salt-leaching technology for human movement monitoring and energy collection has also been investigated (Kapat et al., 2020; Song et al., 2021). Moreover, the fabrication of a calcium phosphate silicate (CPS)-doped PVDF scaffold using a phase-separation-hydration method has shown potential, being mechanically compatible with cancellous bone and encouraging osteoblast redifferentiation (Gong et al., 2022).

The incorporation of other materials has also been explored. For example, PVDF scaffolds doped with zinc oxide nanoparticles (ZnO/PVDF) prepared by electrospinning have shown enhanced mechanical properties and biocompatibility (Li et al., 2018). Similarly, the use of core-shell nano-fibrous membranes made of a polycaprolactone/gelatin core and a poly (vinyl alcohol)/PVDF shell has demonstrated great hemocompatibility, antibacterial activity, and the ability to enhance tissue regeneration (Rahmati et al., 2022).

Poly (hydroxybutyrate-co-hydroxyvalerate) (PHBV) has emerged as a promising material in the field of bone tissue engineering. The development of PHBV/chitosan nanocomposite scaffolds incorporated with Nano-Hydroxyapatite (nHA) has shown potential as an alternative for bone tissue engineering due to enhanced biocompatibility and osteoconductivity (Amaro et al., 2020).

Novel biocomposites providing superior mechanical properties have been developed using PHBV and nano-Hydroxyapatite (nHA). These composites are suggested to mimic the mechanical properties of natural bone, providing further potential for the application of PHBV in bone tissue engineering (Chernozem et al., 2019).

Advancements in technology have enabled the fabrication of bone scaffolds composed of PHBV and nano-Hydroxyapatite (nHA) through 3D printing technology. These scaffolds have been suggested to enhance cell proliferation and osteogenic differentiation, presenting a viable option for bone tissue regeneration (Gorodzha et al., 2017). Further studies have explored the biodegradable material Poly (3-hydroxybutyrate-co-3-hydroxyvalerate) (PHBV) and its use in the construction of functionalized scaffolds. PHBV scaffolds incorporated with bioactive glass nanoparticles could promote osteoblast proliferation and maturation, enhancing bone tissue regeneration (Jiao et al., 2020c).

In conclusion, the use of polymer piezoelectric materials in bone tissue engineering offers promising avenues for effective bone regeneration and repair. As technology advances, the optimization of these materials for specific applications will continue to be a vital area of research.

## 5 Conclusion

Since 2007, the application of piezoelectric materials in the field of orthopedics has gradually received widespread attention, especially in North America, Europe and Asia (Figure 3A). Institutions in these regions have a strong influence in terms of research collaborations and the number of papers published. Next, we analyze the top authors of piezoelectric materials application in orthopedics, whose research results are instructive for the field. LANCEROS-MENDEZ S and RIBEIRO C focus on the design and evaluation of polymer piezoelectric materials (PVDF and PHBV) in orthopedic implants; Zhao Ke and Tang Yifeng mainly research bioceramic piezoelectric materials BaTiO3 and polymer piezoelectric materials PVDF and PHBV. These studies provide important references and guidance for the development of materials in the biomedical field.

According to the keywords, we divided the research content into 17 categories, and summarized five main research directions: 1. Piezoelectric bone surgery in oral and maxillofacial surgery; 2. Piezoelectric bone tissue engineering scaffold; 3. Manufacture for hearing loss patients 4. Physical properties of piezoelectric materials; 5. Using piezoelectric materials as anti-corrosion coatings on the surface of alloy implants. Popular keywords show that piezoelectric bone tissue engineering scaffolds are the latest hot direction in this field. In addition, piezoelectric materials are mainly divided into bioceramic piezoelectric materials and polymer piezoelectric materials, which have a wide range of applications in bone-related fields, such as the preparation of piezoelectric bone tissue engineering scaffolds by 3D printing and non-3D printing, and as alloy implants Anti-corrosion coating on the surface of the object.

Piezoelectric materials, primarily bioceramic and polymer types, have demonstrated significant potential in bone-related applications. Bioceramic piezoelectric materials, such as Barium Titanate (BaTiO3) and perovskite ceramics, have shown promising results in promoting bone formation, osseointegration, and osteogenic activity. Polymer piezoelectric materials, including polyvinylidene fluoride (PVDF) and its composites, have also exhibited excellent biocompatibility, making them attractive for tissue engineering applications. The piezoelectric effect has been investigated in enhancing 3D hydrogel encapsulation, growth factor delivery, and scaffold modification, leading to improved cell in-growth, vascularization, and osteogenic protein levels. Despite concerns about potential toxicity, studies have suggested the safety of BaTiO3-based piezoelectric materials in biomedical applications. Recent advancements in piezoelectric materials have opened up new possibilities in oral implantology and the repair of large bone defects. Further research and optimization of parameters are needed to evaluate the effects of piezoelectric materials with other types of scaffold materials and cells. As technology advances, the optimization of these materials for specific applications will continue to be a vital area of research.
